# Reduction of Lipid-Core Burden Index in Nonculprit Lesions at Follow-Up after ST-Elevation Myocardial Infarction: A Randomized Study of Bioresorbable Vascular Scaffold versus Optimal Medical Therapy

**DOI:** 10.1155/2021/5590093

**Published:** 2021-07-01

**Authors:** Joelle Kefer, Patrick Chenu, Olivier Gurné, Frederic Maes, Théophile Tamakloé, Christophe Beauloye

**Affiliations:** ^1^Division of Cardiology, Cliniques Universitaires Saint-Luc, Université Catholique de Louvain (UCLouvain), Brussels, Belgium; ^2^Pôle de Recherche Cardiovasculaire, Institut de Recherche Expérimentale et Clinique (IREC), Université Catholique de Louvain (UCLouvain), Brussels, Belgium

## Abstract

**Background:**

Non-flow-limiting nonculprit lesions (NCL) that contain a large lipid-rich necrotic core (nonculprit lipid-rich plaques (NC-LRP)) are most likely to cause recurrent acute coronary syndrome after ST-elevation myocardial infarction (STEMI). Near-infrared spectroscopy (NIRS) detects LRPs using the maximum 4 mm lipid-core burden index (maxLCBI_4 mm_). Few data are available regarding NIRS-guided therapy of these NC-LRPs, which are a potential target for preventive stenting. Bioresorbable vascular scaffold (BVS) provides local drug delivery and could facilitate plaque passivation after resorption. This study sought to assess the safety of BVS implantation in NC-LRPs and its efficacy in reducing maxLCBI_4 mm_ at 2-year follow-up after STEMI.

**Methods and Results:**

In total, 33 non-flow-limiting NCLs from 29 STEMI patients were included in this study. Of these, 15 were LRPs and were randomly assigned to either the BVS + optimal medical therapy (OMT) arm (group 1; *N* = 7) or the OMT arm (group 2; *N* = 8). At baseline, there were no differences in plaque characteristics between groups (fractional flow reserve: 0.85 ± 0.04 vs. 0.89 ± 0.06; diameter stenosis (DS): 43.4 ± 8 vs. 40.1 ± 10.7%; plaque burden 54.98 ± 5.8 vs. 49.76 ± 8.31%; and maxLCBI_4 mm_ 402 [348; 564] vs. 373 [298; 516]; *p*=*NS* for all comparisons between groups 1 and 2, respectively). Seven BVSs were implanted 3 ± 1 days after STEMI in six patients, without complications. At angiographic follow-up (712 [657; 740] days), a significant and similar reduction of maxLCBI_4 mm_ was observed in both groups, with a median change of 306 [257; 377] in group 1 vs. 300 [278; 346] in group 2 (*p*=0.44). DS was significantly lower in group 1 vs. group 2 (19.8 ± 7 vs. 41.7 ± 13%, *p*=0.003), while plaque burden remained unchanged in both groups. Overall survival was 100%, target lesion failure was 13%, and stent thrombosis was 0%.

**Conclusions:**

BVS + OMT and OMT appear as similarly safe and effective in reducing maxLCBI_4mm_ in NC-LRPs at 2-year follow-up after STEMI.

## 1. Introduction

Multivessel coronary artery disease is common in patients with ST-elevation myocardial infarction (STEMI) and is associated with worse outcome [1]. Major adverse cardiac events (MACE) after acute coronary syndrome were attributed to non-culprit lesions (NCL) in 11.6% of patients in the PROSPECT trial [2]. NCL-related MACE arose more frequently from lesions with a diameter stenosis (DS) <50%, a thin cap, a large plaque burden, and a large lipid-rich necrotic core (lipid-rich plaque (LRP)). Revascularization by percutaneous coronary angioplasty (PCI) of NCL that are angiographically severe or flow-limiting, as assessed by fractional flow reserve (FFR), has been shown to reduce cardiovascular death or myocardial infarction (MI) after STEMI [3]. Nevertheless, the ability of prophylactic PCI of NCL with a benign angiographic appearance but high-risk morphologic characteristics of vulnerable plaques might mitigate the risk of future cardiac events after STEMI remains under debate. Near-infrared spectroscopy (NIRS) accurately differentiates between lipid-rich and lipid-poor plaque based on the absorption pattern of scattered light in the near-infrared range [4]. NIRS-derived lipid-core burden index in a 4 mm length artery (maxLCBI_4 mm_) has been shown to identify vulnerable plaques and predict NCL-related MACE originating from within the maxLCBI_4 mm_ site [5]. Lesions with both large plaque burden by IVUS and large lipid-rich cores by NIRS had a 4-year higher risk of NCL-related MACE [6]. Bioresorbable vascular scaffold (BVS) provides transient mechanical vessel support and local drug delivery in the coronary wall, with a potential for plaque passivation and vessel remodeling after resorption of the polymeric structure [7]. Intravascular analyses have demonstrated a plaque-media reduction and histological modifications at 5 years after BVS implantations in stenotic coronary segments [8].

This study sought to investigate the safety of BVS implantation in non-flow-limiting nonculprit LRP (NC-LRP) and its efficacy in reducing maxLCBI_4 mm_ at 2-year follow-up after STEMI.

## 2. Methods

### 2.1. Study Design

**Figure 1:**
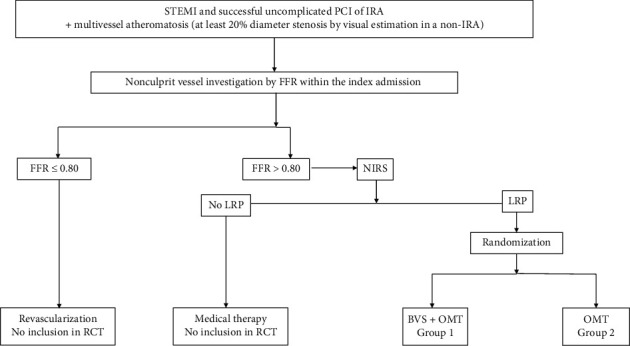
Study design. STEMI = ST-elevation myocardial infarction; PCI = percutaneous coronary intervention; IRA = infarct-related artery; FFR = fractional flow reserve; NIRS = near-infrared spectroscopy; LRP = lipid-rich plaque; RCT = randomized control trial; BVS = bioresorbable vascular scaffold; and OMT = optimal medical therapy.

The study included consecutive STEMI patients who underwent a successful uncomplicated PCI of the infarct-related artery (IRA) in our center between January 2015 and December 2017 and who had a multivessel coronary artery disease with at least 20% DS by visual estimation at angiography in a non-IRA. The nonculprit vessel was investigated by FFR, intravascular ultrasound (IVUS), and NIRS during the index admission in order to detect NC-LRP and consecutively randomize them to one of the following two therapeutic strategies: BVS associated with optimal medical therapy (BVS + OMT = group 1) versus OMT only (group 2). The study design is detailed in Figure 1.

### 2.2. Patient Population

The inclusion criteria were age between 18 and 80 years, STEMI treated by a successful and uneventful PCI (TIMI 3 flow, residual DS < 30%), and multivessel coronary atherosclerosis with a least one NCL (minimum 20% DS by visual estimation in a non-IRA). The exclusion criteria were cardiogenic shock, left main disease, prior coronary bypass graft surgery, left ventricular ejection fraction <35%, severe renal failure (glomerular filtration rate <30 ml/min/m^2^), contraindication for dual antiplatelet therapy, need for noncardiac surgery within 1 year, woman with probability of pregnancy, and impossibility or unwillingness to sign the informed consent.

The protocol was approved by the Ethics Committee (Comité d'Ethique Hospital-Facultaire de l'Université Catholique de Louvain: 2014/3OCT/498).

### 2.3. Cardiac Catheterization

The non-culprit vessel was deemed appropriate if it had a reference vessel diameter (RVD) ≥2.5 mm assessed by quantitative coronary analysis (QCA), was free of previous PCI, and was suitable for investigation by FFR and NIRS catheter, performed during the index admission. For each lesion, RVD, minimum luminal diameter (MLD), and DS were assessed by two readers by QCA using the CAAS system (Pie Medical Imaging, the Netherlands).

The FFR was determined using the Navvus system (ACIST Medical Systems, Eden Prairie, MN, USA), a rapid-exchange microcatheter with fiber-optic sensor placed distally to the target lesion; after intracoronary injection of 0.5 mg isosorbide dinitrate, the FFR was measured under hyperemia induced by intravenous infusion of adenosine at 140 *μ*g/kg/min.

In case of FFR ≤ 0.80, the nonculprit vessel was treated by revascularization and was not considered for randomization. If the FFR was >0.80, the nonculprit vessel was investigated by the NIRS catheter.

### 2.4. Near-Infrared Spectroscopy and Intravascular Ultrasound Imaging Technique

The NIRS system consists of a 3.2 F rapid-exchange catheter (TVC Insight Imaging System, Infraredx, Burlington, MA, USA), a pull-back and rotation device, and a console. The catheter provides a multimodality investigation of the vessel, with coregistered intravascular ultrasound data and near-infrared laser imaging. The catheter was advanced along a 0.014-inch guidewire into the investigated vessel, distally to the target lesion. IVUS data were acquired during an automated pull-back, at a speed of 0.5 mm/s and 16 rotations per second. The IVUS measurements were performed according to the relevant expert consensus document [9] in order to acquire the minimum lumen area (MLA) and plaque burden (calculated as vessel area – lumen area/vessel area). Calcium deposits were graded semiquantitatively as absent or subtending 1, 2, 3, or 4 quadrants. An off-line analysis was performed using the TVC Insight Imaging System software (Infraredx, Burlington, MA, USA).

The NIRS examination uses absorbance and backscattering of the near-infrared wavelength to provide a chemogram that is correlated with the histological composition of the investigated tissue [4]. Every measurement of the chemogram is binary coded as yellow (positive) or red (negative). A lipid-core burden index (LCBI) is provided as the sum of the lipid signals along the interrogated vessel segment on a scale of 0 to 1,000. The LCBI is calculated as a fraction of yellow pixels in the chemogram multiplied by a factor of 1,000. The maxLCBI_4 mm_ corresponds to the maximum LCBI in a 4 mm length of the artery. The NIRS probe at the distal tip of the catheter acquires 40 spectroscopic measurements per second during the same pull-back as the IVUS probe.

LRP was defined as an atheromatous segment of minimum 10 mm length containing a chemogram showing a maxLCBI_4 mm_ > 250.

MLA and plaque burden were measured at the site of the target NC-LRP, with the analysis performed by two readers.

**Figure 2:**
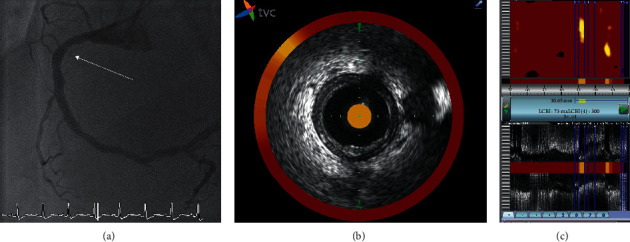
Example of an NIRS and IVUS investigation. (a) Angiogram of a nonculprit lesion located on the right coronary artery. The arrow shows the side branch, landmark for the IVUS analysis. (b) IVUS tomographic image of the right coronary artery obtained at the site of the side branch, showing a noncalcified atheroma. (c) NIRS chemogram from this lesion demonstrating the presence of a lipid-rich plaque (maxLCBI_4 mm_ measured at 300). IVUS = intravascular ultrasound; NIRS = near-infrared spectroscopy; and maxLCBI_4 mm_ = maximum lipid-core burden index in a 4 mm length of the artery.

Figure 2 shows an example of an NIRS chemogram and the coregistered IVUS image of an LRP.

### 2.5. Randomization of Therapeutic Strategies

When at least one segment of minimum 10 mm length containing an NCL with an FFR > 0.80 and a chemogram showing a maxLCBI_4 mm_ > 250, the plaque was randomly assigned to be treated by either BVS + OMT (group 1) or OMT alone (group 2).

OMT consisted of a statin for reducing low-density lipoprotein- (LDL-) cholesterol, lifelong low-dose aspirin, a thienopyridine for at least 12 months, an angiotensin-converting enzyme (ACE) inhibitor (or angiotensin-II receptor blocker, in case of intolerance), and beta-blockers if needed, according to the guidelines [10].

Due to its specific mechanical properties, the implantation of a BVS should follow strict rules (“PSP” score) in order to reach a full expansion of the platform: lesion preparation by balloon predilation, proper vessel and device sizing, prolonged time of inflation during deployment, and postdilation using a noncompliant balloon [11]. The use of intracoronary imaging is strongly recommended for accurate sizing and assessment of the BVS implantation acute result. In the present study, all BVS implantations were performed according to the PSP score and guided by optical coherence tomography (OCT).

OCT was performed with the C7 Dragonfly catheter (St. Jude Medical, Abbott, Santa Clara, CA, USA), which was introduced in the vessel on a 0.014 wire, and provided a series of very high-resolution (15 *μ*m) tomographic images of the coronary segment, using an infrared light technology. During image acquisition, contrast medium is injected at a speed of 3–5 ml/s. With a pullback speed of 10–40 mm/s, image acquisition usually takes 5–10 seconds. The Ilumien console (St. Jude Medical, Abbott, Santa Clara, CA, USA) enables automatic detection of luminal borders as well as online 3D reconstruction, which is helpful to assess the vessel size, appropriate expansion and apposition of the BVS, and absence of acute complication after implantation.

### 2.6. Follow-Up

Clinical follow-up was performed by review of medical records or patient contact by telephone. MACE (death, MI, and need for target lesion revascularization) were documented in order to define target lesion failure (TLF). Only MACEs originating from an NCL have been counted for the secondary endpoint of the study. Antithrombotic and lipid-lowering medications as well as LDL-cholesterol values were recorded at discharge and at follow-up.

Angiographic follow-up was performed at 2 years, with an investigation of the nonculprit vessel by FFR, IVUS, and NIRS in all cases, while OCT was restricted to patients undergoing a BVS implantation. At follow-up, the measurements of plaque burden, MLA, and maxLCBI_4 mm_ were repeated in the same segment, at the site of the original target NC-LRP (in the scaffold for plaques randomized in the BVS + OMT group and in the native vessel for those randomized in the OMT group).

### 2.7. Study Endpoints

The primary endpoint was the change in maxLCBI_4 mm_ at 2-year follow-up. The secondary endpoints were safety of BVS implantation, MACE from an NCL, and TLF at 2-year follow-up.

### 2.8. Statistical Analysis

Continuous variables are presented as mean ± standard deviation when normally distributed and as median and range when nonnormally distributed. Normality was assessed using the Shapiro–Wilk test. Categorical variables are presented as counts and percentages. Continuous variables were tested using the independent samples *t*-test and categorical variables using chi-square or Fisher's exact test, when appropriate.

The interobserver agreement on the measurements of MLD, RVD, and DS by QCA, and MLA and plaque burden by IVUS was expressed as intraclass coefficient. A *p* value <0.05 was considered statistically significant. Analyses were performed using the XLSTAT software (version 2021, Addinsoft, France).

## 3. Results

### 3.1. Patients

**Figure 3:**
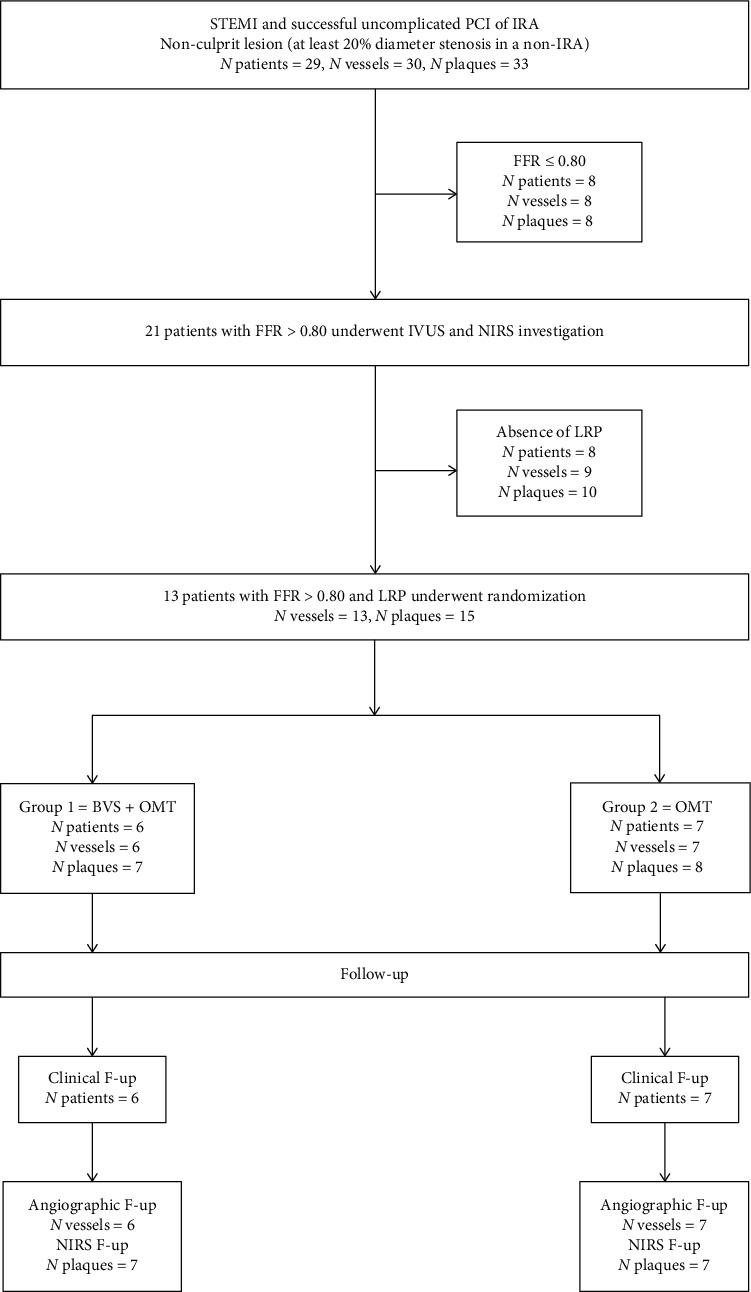
Study flowchart. STEMI = ST-elevation myocardial infarction; PCI = percutaneous coronary intervention; IRA = infarct-related artery; FFR = fractional flow reserve; IVUS = intravascular ultrasound; NIRS = near-infrared spectroscopy; LRP = lipid-rich plaque; BVS = bioresorbable vascular scaffold; and OMT = optimal medical therapy.

Between January 2015 and December 2017, 29 patients were enrolled in the study undergoing a repeated coronary angiography including FFR and NIRS investigation, 3 ± 1 days after a successful and uneventful primary PCI for STEMI. Among them, 13 with hemodynamically non-flow-limiting NCLs containing an LRP were randomized to be treated by BVS + OMT (Group 1, *N* = 6) versus OMT alone (Group 2, *N* = 7). The study flowchart is detailed in Figure 3.

At baseline, patient clinical characteristics were similar between groups (Table 1). Most of them were males (92%), smokers (77%), and not statin users (85%), with a mean age of 56 ± 12 years.

Clinical follow-up was complete in 100% of patients (median duration 762 [712; 1055] days). Survival was 100%, two target lesion revascularizations were observed (one in each group), and no scaffold thrombosis occurred. Overall TLF was 13% and did not differ between groups. At the last follow-up, all but one patient were statin users and the LDL-cholesterol dropped from 107 ± 29 to 64 ± 20 mg/dl, with no differences between Group 1 and Group 2 (68 ± 20 vs. 64 ± 16, resp.; *p*=0.10).

### 3.2. Angiography and Intravascular Imaging

Coronary angiography with NIRS investigation identified 15 LRP in 13 patients during the index admission at 3 ± 1 days after STEMI, without any complication. The angiographic characteristics are detailed in Table 2.

The intraclass correlation coefficients for the measurements of RVD, MLD, DS, MLA, and plaque burden were 0.73, 0.74, 0.86, 0.89, and 0.75, respectively.

At baseline, there was no difference between Groups 1 and 2 in angiographic characteristics (DS 43.4 ± 8 vs. 40.1 ± 10%, resp.; *p*=0.27) or IVUS findings (plaque burden 54.98 ± 5.76 vs. 49.76 ± 8.31%, resp.; *p*=0.10). The vast majority of the coronary plaques (73%) were not calcified. The maxLCBI_4 mm_ was similarly high in both groups (402 [348; 564] vs. 373 [298; 516], respectively; *p*=0.34).

In Group 1, seven BVS were implanted to scaffold seven NC-LRP in six patients. The device diameter was 3.0 mm in six cases and 2.5 mm in one case. The device length was 28 mm in four cases, 18 mm in two cases, and 23 mm in one case. An overlap of two scaffolds was done in one case to cover a very long segment. All lesions were predilated using a semicompliant balloon, all but one BVS were postdilated using a noncompliant balloon, and all implantations were OCT-guided in order to verify the good apposition and complete expansion of the scaffold. There were no periprocedural complications of these BVS implantations.

All patients underwent an angiography at follow-up (median time 712 [657; 740] days) but one of them was not investigated by NIRS; therefore, the complete NIRS follow-up was available for 14/15 coronary plaques (93%).

**Figure 4:**
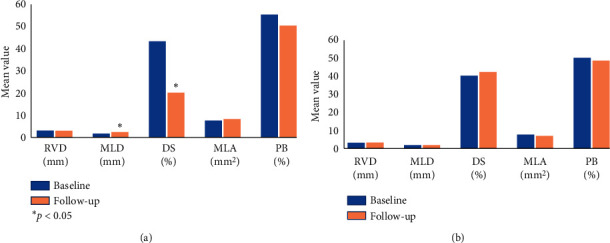
Comparison of baseline and follow-up QCA and IVUS values. (a) Comparison of values in group 1. (b) Comparison of values in group 2. QCA = quantitative coronary angiography; IVUS = intravascular ultrasound; RVD = reference vessel diameter; MLD = minimum lumen diameter; DS = diameter stenosis; MLA = minimum lumen area; and PB = plaque burden.

At follow-up, the DS became significantly lower in the BVS implantation group (19.8 ± 7%, *p* < 0.001 for the comparison vs. baseline), while it remained unchanged under OMT (41.7 ± 13%, *p*=0.34 vs. baseline; *p*=0.003 between groups). The MLA and plaque burden remained unchanged over time in both groups.  Figure 4 shows the comparison of QCA and IVUS values obtained at baseline and follow-up.

**Figure 5:**
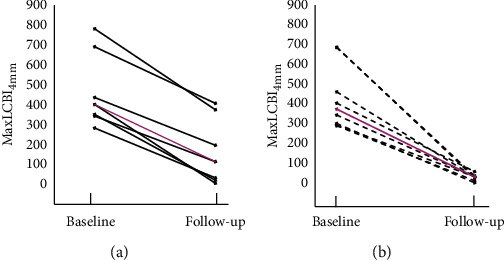
Individual maxLCBI_4 mm_ values at baseline and at follow-up. (a) Values in group 1: black lines are the individual values, and pink line represents the median value of the group. (b) Values in group 2: black lines are the individual values, and pink line represents the median value of the group. BVS = bioresorbable vascular scaffold; OMT = optimal medical therapy; and maxLCBI_4 mm_ = maximum lipid-core burden index in a 4 mm length of the artery.

A significant reduction of the maxLCBI_4 mm_ was observed in both groups (Figure 5): the change of maxLCBI_4 mm_ was 306 [257; 377] in group 1 and 300 [278; 346] in group 2 (*p*=0.44).

At follow-up, a persistently high maxLCBI_4 mm_ of >250 was observed in two cases in Group 1, while in none in Group 2. These two plaques with persistently high lipid component at follow-up after BVS implantation were more calcified at baseline, while all other less calcified NC-LRPs exhibited a decreased maxLCBI_4 mm_ value of <250 after scaffolding. In Group 2, NC-LRP had evolved equally under OMT, regardless of the calcified content.

**Figure 6:**
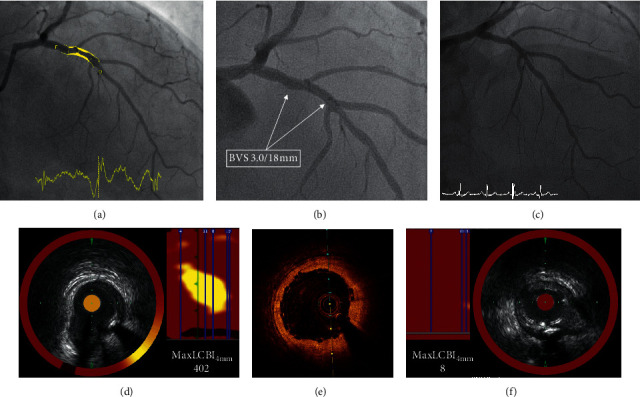
Example of reduction of maxLCBI_4 mm_ after BVS implantation. (a) Baseline angiography showing a moderate lesion in the mid-left anterior descending coronary artery. (b) Angiography of the BVS implantation. (c) Follow-up angiography. (d) Baseline NIRS showing a noncalcified NC-LRP. (e) OCT showing a good acute result of the BVS implantation, with an appropriate expansion and apposition of the scaffold. (f) Follow-up NIRS showing a reduction of maxLCBI_4 mm_ at the site of the original NC-LRP. BVS = bioresorbable vascular scaffold; NIRS = near-infrared spectroscopy; NC-LRP = nonculprit lipid-rich plaque; OCT = optical coherence tomography; and maxLCBI_4 mm_ = maximum lipid-core burden index in a 4 mm length of the artery.

**Figure 7:**
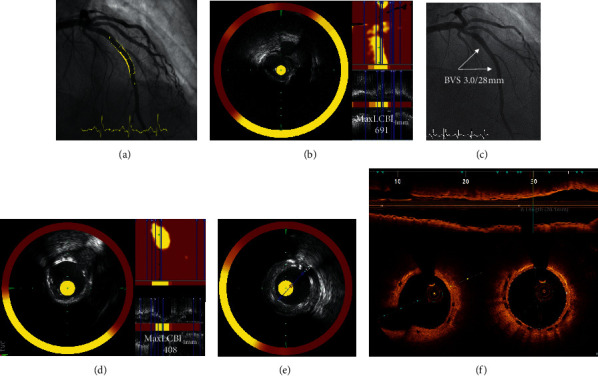
Example of persistently high maxLCBI_4 mm_ after BVS implantation. (a) Baseline angiography showing a moderate lesion in the mid-left anterior descending coronary artery. (b) Baseline NIRS showing a calcified NC-LRP. (c) Angiography of the BVS implantation. (d) Follow-up NIRS showing a persistently high maxLCBI_4 mm_ at the site of the original NC-LRP. (e) IVUS demonstrating good expansion of the scaffold in the calcified plaque. (f) OCT showing a good result of the BVS at follow-up with an appropriate expansion, apposition, and endothelialization. BVS = bioresorbable vascular scaffold; NIRS = near-infrared spectroscopy; NC-LRP = nonculprit lipid-rich plaque; IVUS = intravascular ultrasound; OCT = optical coherence tomography; and maxLCBI_4 mm_ = maximum lipid-core burden index in a 4 mm length of the artery.

Figure 6 illustrates the reduction of maxLCBI_4 mm_ in a low calcified NC-LRP, while Figure 7 shows the persistently high maxLCBI_4 mm_ in a calcified NC-LRP at follow-up after BVS implantation.

## 4. Discussion

### 4.1. Our Findings Suggest Two Hypotheses

1. BVS and OMT appear as effective in reducing maxLCBI_4 mm_ in non-flow-limiting NC-LRP at 2-year follow-up after STEMI
2. BVS implantation in these NC-LRPs after STEMI seems to be performed safely in selected cases, up to 2-year follow-up

### 4.2. Reduction of maxLCBI_4 mm_

Regression of coronary atherosclerosis was first demonstrated in the ASTEROID trial [12] with high-dose rosuvastatin, which induced a 6.8% reduction in total atheroma volume of the human coronary plaque, investigated by IVUS. Moreover, a modification in coronary atheroma composition was observed by spectral analysis of the radiofrequency IVUS signal under high-dose atorvastatin and rosuvastatin [13], consisting in a reduction of estimated fibrofatty tissue volume with atheroma regression, while calcium tissue volume increased. The concept of atheromatous plaque passivation has been defined as a process by which the structure or content of the vulnerable plaque is changed to reduce the risk of subsequent rupture and thrombosis. In the PROSPECT trial [2], vulnerable coronary plaques were characterized by IVUS as thin-cap fibroatheroma with a large plaque burden (≥70%) and small MLA (≤4 mm^2^), and they were identified as predictors of NCL-related events. In the PROPECT-II study [6], MACE occurred in 13% patients within 4 years after myocardial infarction, with 8% arising from untreated NCL, while 4% from culprit lesions. Most events arose from untreated angiographically mild lesions, nonhemodynamically significant, which had high lipid content (maxLCBI_4 mm_ > 324) and large plaque burden (≥70%). These observations may support studies considering invasive treatment of this kind of vulnerable high-risk coronary plaques.

Preventive BVS implantation in nonobstructive lesions with a plaque burden >65% has been shown to induce an enlarged MLA with a favorable clinical outcome [14].

Plaque passivation has been described as one of the most interesting promises of BVS in the long term [15]: in addition to plaque volume stabilization [16], a reduction in neointimal macrophage infiltration and overall lipid content was suggested by OCT follow-up images, performed after resorption of the scaffold. Unlike metallic stents, BVS may compensate the lumen narrowing induced by neointimal capping due to expansive remodeling and late lumen enlargement. [17]. The LRP study [5] showed that the ability of NIRS to predict event-prone plaques appears to be independent of plaque burden or MLA within the site of maxLCBI_4 mm_; in nonobstructive plaques with a maxLCBI_4 mm_ ≥ 250, the hazard ratio for future events was 1.21 for each 100-unit increase of maxLCBI_4 mm_. The lipid content, identified by NIRS investigation, could therefore be considered as a target for assessing vulnerable plaque passivation after treatment. In addition, the maxLCBI_4 mm_ is an indicator that is more easily and quickly obtained than MLA and plaque burden, which require core labs or teams very experienced in IVUS for accurate and reproducible assessment.

In the present study, the objective was to compare two therapeutic strategies (OMT vs. BVS + OMT) and their impact on the modification of this index, which is highly predictive of NCL-related events. We observed a significant and similar change in maxLCBI_4 mm_ after 2 years in both strategies. OMT, including high doses of statin, led to a reduction of LDL-cholesterol at 64 ± 20 mg/dl.

In the PROSPECT ABSORB study [14], the maxLCBI_4 mm_ remained high under medical therapy (from 337.2 to 268.8), while it dropped significantly after BVS (from 323.6 to 62; *p* < 0.0001). Conversely, our results showed that maxLCBI_4 mm_ significantly dropped in NC-LRPs after 2 years of medical treatment (from 373 to 31), this reduction not being significantly different from that observed after BVS (from 402 to 116; *p*=0.10). These discrepancies could be explained by the lower number of patients under high-dose statin at follow-up (84 vs. 92%) and by the different plaque characteristics at baseline (plaque burden: 73.7 vs. 52.2%; MLA 2.9 vs. 7.57 mm^2^) in the PROSPECT ABSORB vs. our study, respectively.

In our study, a reduction of maxLCBI_4 mm_ was observed in all plaques. However, a persistently high value of maxLCBI_4 mm_ > 250 was observed in two cases after BVS implantation, while in none under medical therapy only. This could be partly explained by the higher calcium content of these two plaques. In the PROSPECT ABSORB study [14], a high maxLCBI_4 mm_ of >324 was also found in 19% of plaques at follow-up after BVS.

These findings require further investigations in larger studies so as to identify which characteristics of the coronary plaques are predictive of bioresorbable stent-induced plaque passivation.

### 4.3. Safety of BVS Implantation in Nonculprit Lesions

Prophylactic stenting of non-flow-limiting lesions with benign angiographic appearance for passivation of nonculprit vulnerable plaques remains controversial. Stent or BVS implantation in such lesions may theoretically result in periprocedural complications related to fibrous cap rupture, leading to lipid-rich core distal embolization and periprocedural MI. In the COLOR study [18], complications of PCI with drug-eluting stents of NIRS-identified LRP occurred in 0.45% of cases, including one slow flow, four dissections, one MI, and one emergent coronary bypass.

In the long term, the risk of restenosis and stent/scaffold thrombosis may be increased when the implantation occurs in this prothrombotic post-STEMI phase.

In our study, BVS implantation was safe, with no periprocedural complications, no scaffold thrombosis at follow-up, one single case of target lesion revascularization for restenosis, no deaths, and no late strut maloppositions or fractures at OCT follow-up. This outcome is better than that in previously published series on BVS implantation [7, 19]. Possible explanations could be that, in the present study, BVS was implanted in nonstenotic, few calcified and soft lesions, enabling an easier complete expansion of the scaffold than in stenotic calcified vessels. Moreover, BVS implantations were performed under OCT guidance, according to the rules of the PSP score, thereby ensuring optimal expansion and complete apposition of the scaffold and the absence of acute angiographic complications, factors that likely lower the risk of early and late thrombosis and restenosis [11, 20]. These technical aspects are of paramount importance to allow the bioresorbable scaffold acting as a vascular reparative therapy, i.e., generating a neocap of intimal hyperplasia, delivering the drug locally, and then resorbing, in order to achieve a passivation of the vulnerable plaque and ultimately reduce the NCL-related events.

## 5. Conclusion

This study suggests that both OMT alone and BVS associated with OMT are safe and effective in reducing maxLCBI_4 mm_ in NC-LRP up to 2 years after STEMI. Larger studies are needed in the future in order to confirm this pilot study and better identify the type of plaques better responding to the bioresorbable stent-induced passivation process.

## Figures and Tables

**Table 1:** Patient's characteristics.

Characteristics		All	Group 1	Group 2	*p* value
			BVS + OMT	OMT	
		*N* patients = 13	*N* patients = 6	*N* patients = 7	
Age (years)	Mean ± sd	56 ± 12	57 ± 11	56 ± 11	0.36
Male gender	*n* (%)	12 (92)	5 (83)	7 (100)	0.46
Smoking	*n* (%)	10 (77)	5 (83)	5 (71)	1
Hypertension	*n* (%)	6 (46)	3 (50)	3 (43)	0.61
Diabetes	*n* (%)	3 (23)	1 (17)	2 (28)	1
Glomerular filtration rate (ml/min/1.73 m^2^)	Mean ± sd	92 ± 12	90 ± 14	97 ± 5	0.12
Total cholesterol (mg/dl)	Mean ± sd	176 ± 33	166 ± 26	184 ± 35	0.16
LDL cholesterol (mg/dl)	Mean ± sd	107 ± 29	94 ± 13	118 ± 32	0.06
HDL cholesterol (mg/dl)	Mean ± sd	37 ± 8	37 ± 7	38 ± 8	0.38
Statin user	*n* (%)	2 (15)	1 (17)	1 (14)	1

**Table 2:** Angiographic characteristics.

Characteristics		All	Group 1	Group 2	*p* value
			BVS + OMT	OMT	
		*N* plaques = 15	*N* plaques = 7	*N* plaques = 8	
*Baseline*					
*Coronary artery segment location*					
Proximal left anterior descending	*n* (%)	2 (13)	1 (14)	1 (12)	NS
Mid-left anterior descending	*n* (%)	6 (40)	4 (57)	2 (25)	NS
Distal left anterior descending	*n* (%)	1 (6)	1 (28)	0	NS
Distal left circumflex	*n* (%)	2 (13)	0	2 (25)	NS
Proximal right	*n* (%)	1 (6)	0	1 (12)	NS
Mid-right	*n* (%)	3 (20)	1 (28)	2 (25)	NS

*Quantitative Coronary Angiography*					
Reference vessel diameter (mm)	Mean ± sd	3.09 ± 0.55	3.05 ± 0.32	3.12 ± 0.65	0.4
Minimum lumen diameter (mm)	Mean ± sd	1.79 ± 0.43	1.71 ± 0.37	1.85 ± 0.44	0.28
Diameter stenosis (%)	Mean ± sd	41.6 ± 10	43.4 ± 8	40.1 ± 10.7	0.27
*Fractional flow reserve*	Mean ± sd	0.87 ± 0.06	0.85 ± 0.04	0.89 ± 0.06	0.13

*IVUS findings*					
Minimum lumen area (mm^2^)	Mean ± sd	7.57 ± 2.33	7.50 ± 0.90	7.63 ± 2.96	0.45
Plaque burden (%)	Mean ± sd	52.2 ± 7.96	54.98 ± 5.76	49.76 ± 8.31	0.1

*Arc of calcium at the LRP site*					
0 quadrants	*n* (%)	11 (73)	5 (71)	6 (75)	NS
1 quadrant	*n*	2 (13)	1	1	NS
2 quadrants	*n*	1 (6)	0	1	NS
3 quadrants	*n*	1 (6)	1	0	NS
4 quadrants	*n*	0	0	0	NS

*NIRS findings*					
MaxLCBI_4mm_	Median (*Q*1, *Q*3)	402 (321, 572)	402 (348, 564)	373 (298, 516)	0.34

*Follow-up*					
*Quantitative Coronary Angiography*					
Reference vessel diameter (mm)	Mean ± sd	3.07 ± 0.55	2.96 ± 0.36	3.17 ± 0.63	0.25
Minimum lumen diameter (mm)	Mean ± sd	2.06 ± 0.46	2.35 ± 0.30	1.81 ± 0.38	0.01
Diameter stenosis (%)	Mean ± sd	31.6 ± 16.1	19.8 ± 7.4	41.7 ± 13.2	0.003
Fractional flow reserve	Mean ± sd	0.90 ± 0.05	0.88 ± 0.04	0.94 ± 0.02	0.18

*IVUS findings*					
Minimum lumen area (%)	Mean ± sd	7.57 ± 2.12	8.17 ± 1.34	6.84 ± 2.42	0.22
Plaque burden (%)	Mean ± sd	49.13 ± 8.50	50.02 ± 9.37	48.24 ± 6.95	0.1
Change of plaque burden	Mean ± sd	2.55 ± 10.65	5.83 ± 6.87	1.39 ± 11.92	0.14

*NIRS findings*					
MaxLCBI_4mm_	Median (*Q*1, *Q*3)	31 (17, 127)	116.5 (26, 331)	31 (11, 31)	0.1
Change of maxLCBI_4mm_	Median (*Q*1, *Q*3)	300 (269, 370)	306 (257, 377)	300 (278, 346)	0.44

## Data Availability

The data used to support the findings of this study are available from the corresponding author upon request.

## Additional Points

This was a pilot study performed on a small number of patients and plaques, in a single center. IVUS, NIRS, and QCA analyses were not performed by a core lab but by two investigators (JK and TT), with evaluation of the interobserver agreement.
